# Torsion of the appendix testis in an undescended testicle

**DOI:** 10.11604/pamj.2015.22.265.7493

**Published:** 2015-11-20

**Authors:** Fouad Hajji, Ahmed Ameur

**Affiliations:** 1 Department of Urology, Mohammed V Military University Hospital, 10100 Rabat, Morocco

**Keywords:** Torsion, appendix testis, undescended testis, cryptorchidism

## Image in medicine

A 21-year-old man with a history of left uncorrected cryptorchidism presented to the Emergency Department with sudden onset of left groin pain. He did not have any nausea, vomiting, or fevers. Additionally, there was no history of trauma, change in bowel habits or genitourinary symptoms prior to the onset of the pain. Physical examination revealed a tender, swollen and painful undescended testis located in the inguinal area with an empty ipsilateral hemiscrotum. Men presenting with pain and undescended testis are more likely to have testicular torsion, malignancy, associated inguinal hernia or trauma. The extra-scrotal position of the testis expands the differential diagnosis to also include suppurative inguinal lymphadenitis, fat necrosis, impacted ureteral stone or epididymitis. Doppler examination; however, was not performed in our patient so as to avoid unnecessary delay in the surgical exploration of the groin to potentially avert missed testicular torsion or incarcerated hernia. Intra-operative findings revealed torsion of the appendix testis in an inguinal cryptorchid testicle. The spermatic cord was untwisted and no associated hernia sac was identified. The necrotic appendix was excised and cryptorchidism corrected. Torsion of the appendix testis in an undescended testis is an uncommon condition. Although it can be managed conservatively, the diagnosis is significantly more challenging than in normally descended testes. Hence, it should reduce the threshold for surgical exploration to clear up any doubts. Excision of the twisted appendix can rapidly relieve refractory pain, avert reactive epididymitis as well as hasten recovery, which seems to have happened in our patient.

**Figure 1:**
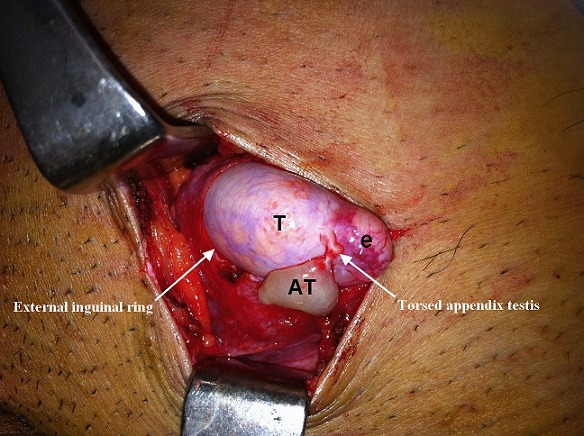
Torsion of the appendix Testis in an undescended testicle. (AT): appendix testis; (T): testis; (e): epididymis

